# Effects of one week different intensity endurance exercise on cardiorespiratory and cardiometabolic markers in junior young athletes

**DOI:** 10.1136/bmjsem-2019-000644

**Published:** 2019-09-18

**Authors:** Gashaw Tesema, Mala George, Soumitra Mondal, D Mathivana

**Affiliations:** 1 Department of Sport Science, College of Natural and Computational science, Mizan-Tepi University, Tepi, Ethiopia; 2 Department of Sport Science, College of Natural and Computational Sciences, Mekelle University, Mekelle, Ethiopia; 3 Department of Biochemistry, College of Health Sciences, Mekelle University, Mekelle, Tigray, Ethiopia; 4 Department of Sport science, College of Natural and Computational Sciences, Mekelle University, Mekelle, Tigray, Ethiopia

**Keywords:** cardiorespiratory markers, cardio-metabolic markers, training intensities, young athletes, Heart rate drift

## Abstract

**Background:**

Endurance training in varied intensity zones are more likely to show different adaptive responses. However, it is still unclear the intensity level of endurance exercise in junior young athletes that may promote increased markers for cardiorespiratory and cardiometabolic response. Therefore, we aimed to investigate the effect of 1 week different intensity endurance training on markers of cardiorespiratory and cardiometabolic response in young athletes.

**Methods:**

Thirty apparently healthy male young athletes averaging 19.4±1.3 years of age were recruited and randomly assigned to two different intensity endurance training groups. The first group trained at 60% to 70% maximum heart rate (HRmax) for 45 min per session and the second group trained at 70% to 80% HRmax for 30 min per session for a period of 1 week three sessions per week. A pre-training and post-training, marker of cardiorespiratory and cardiometabolic response was assessed for both groups.

**Results:**

Both intensity training had no significant effect on markers of cardiorespiratory response within and between training groups (p>0.05). Whereas, cardiometabolic markers lactate dehydrogenase (p=0.001), cardiac troponin I (p=0.001) and creatine kinase myocardial band (p=0.001) significantly increased at the end of 1 week within the group in both training groups (p≤0. 05). In the comparative analysis between groups, there was no significant difference in markers of cardiometabolic response (p>0.05).

**Conclusion:**

1 week 60% to 70% HRmax and 70% to 80% HRmax intensity zones induces similar acute effects on markers for cardiometabolic and cardiorespiratory responses between groups and significant elevations of cardiometabolic markers within the groups in moderately trained young athletes.

**Key messages**

**What are the new findings**

- Different endurance training intensity zones (60% to 70% maximum heart rate (HRmax) and 70% to 80% HRmax) responses similar acute effects on cardiometabolic and cardiorespiratory markers.
- Elevations of serum cardiometabolic markers have been observed similarly on both intensity zones in young athletes.

**How might it impact on clinical practice in the near future**

- Evaluation of cardiometabolic markers of athletes during clinical evaluation might be false-positive which need careful assessment using different measurement evaluations.

## Introduction

Endurance events are becoming increasingly popular^1^ both in young and recreational athletes. Although regular physical activity exerts a range of beneficial physiological effects on cardiovascular health, but a long-duration ultra-endurance exercise might produce substantial changes in cardiometabolic parameters^2 3^ and muscle damage.^4^ The research published so far reported several result differences for many reasons, such as the variation in the duration and intensity of the physical exercises.^5^ In addition, studies involving intensifications of training in already well-trained athletes have shown equivocal results.^5 6^ However, little information is available on the effects of different intensities, in the modulation of the markers for cardiorespiratory and cardiometabolic stress especially in young moderately trained athletes. Therefore, such knowledge could help young and recreational athletes create effective exercise programmes for muscle strength and cardiorespiratory adaptation.

The optimum training intensity for eliciting maximum performance adaptation in endurance exercise at steady state remains inconclusive. Effect of high intensity exercise on skeletal and cardiac muscle cells results into increased metabolic stress markers.^7 8^ Thus, higher values than normal serum cardiac stress markers like cardiac troponin I (cTnI),^9 10^ creatine kinase myocardial band (CK-MB) and lactate dehydrogenase (LDH),^11 12^ have been reported. Interestingly, a study by Peake *et al*^13^ found out that, there were significant elevation in serum cardiac markers in response to both moderate and high intensity training. However, studies by Daussin *et al*, Helgerud *et al* and Gorostiaga *et al*,^14–16^ reported no difference in the increase in maximum oxygen consumption (VO_2_max) between high and moderate intensity exercise training. Therefore, the above inconclusive findings could be due to either smaller or bigger differences in exercise intensities. Hence, endurance training in varied intensity zones, are more likely to show different adaptive responses.

It is still unclear the intensity level of endurance exercise that may promote increased markers for cardiorespiratory and cardiometabolic after short-term exposure to endurance exercise training in moderately trained athletes. Therefore, in this study, we aimed to observe the immediate effect at recovery after 1 week different intensity endurance training on cardiorespiratory and cardiometabolic markers of young athletes. Hence, we applied 1 week endurance training 60% to 70% maximum heart rate (HRmax) for 45 min per session for the first group and 70% to 80% HRmax for 30 min per session for a period of 1 week for the second group for 3days per week^-1^. The duration was as it is recommended for improving and maintaining cardiorespiratory fitness for health purposes.^17^

## Methods

### Study area

This study was conducted in Bahir Dar, located about 578 km north-northwest of Addis Ababa, Ethiopia. It has an altitude of 1840 metres above the sea level and within latitude and longitude of 11°36′N 37°23′E coordinates, while the annual average temperature is 25°C to 32°C, with an average humidity of 58%.^18^

### Participants and sample size

Thirty apparently healthy male young athletes within 19.4±1.3 years of age were recruited from Bahir Dar University Sport Academy. All participants were moderately trained and active in involving in ball games and athletics trainings given in the sport academy and familiar to a workload of 3 days per week.

The sample size was based on similar studies done before^19^ using the following equations^20^ which considers 80% power and p≤0.05. Where, n=sample size needed per group, σ^∗2^ T is variance of the treatment, σ^∗2^ R is variance of the reference, δ is the maximum percent of no clinical importance, CVT is coefficient of variations of the treatment, CVR is coefficient of variations of the reference.

$$n=\frac{{\left(z\alpha +z\beta /2\right)}^{2}\left(\sigma 2+\sigma 2\right)R*T*}{{\left(\delta -/CVT-CVR/\right)}^{2}},n=\frac{{\left(1.64+0.84\right)}^{2}\left(0.30^{2}+0.35^{2}\right)}{{\left(0.10+0.70-0.50\right)}^{2}}=14.5$$

Consequently, a validated Physical Activity Readiness Questionnaire^21^ was used to evaluate conditions that may prohibit participants from practising in endurance training.

### Inclusion and exclusion criteria

Participants that reported problems in their health (heart dysfunction and chest pain during exercise) and physical conditions (loss of balance, consciousness and bone or joint problem) and are currently taking antihypertensive drugs were excluded from the study. In addition, smokers and alcoholics were also excluded from participating in the study due to reported influences on both metabolic and physical performance biomarkers.^22 23^ Participants with age 18 to 25 years of old were included. This is due to the reason that fitness of sports men above 25 years becomes decline and fitness of youngsters with age below 18 years is in the process of growing which can be confounding factors for the result. Only male athletes also included in the study for maintaining homogeneity of the experiment, moderately trained athletes, who fulfils the Physical Activity Readiness Questionnaires and passed the aforementioned exclusion criteria were included in the study.

### Study design

**Figure 1:**
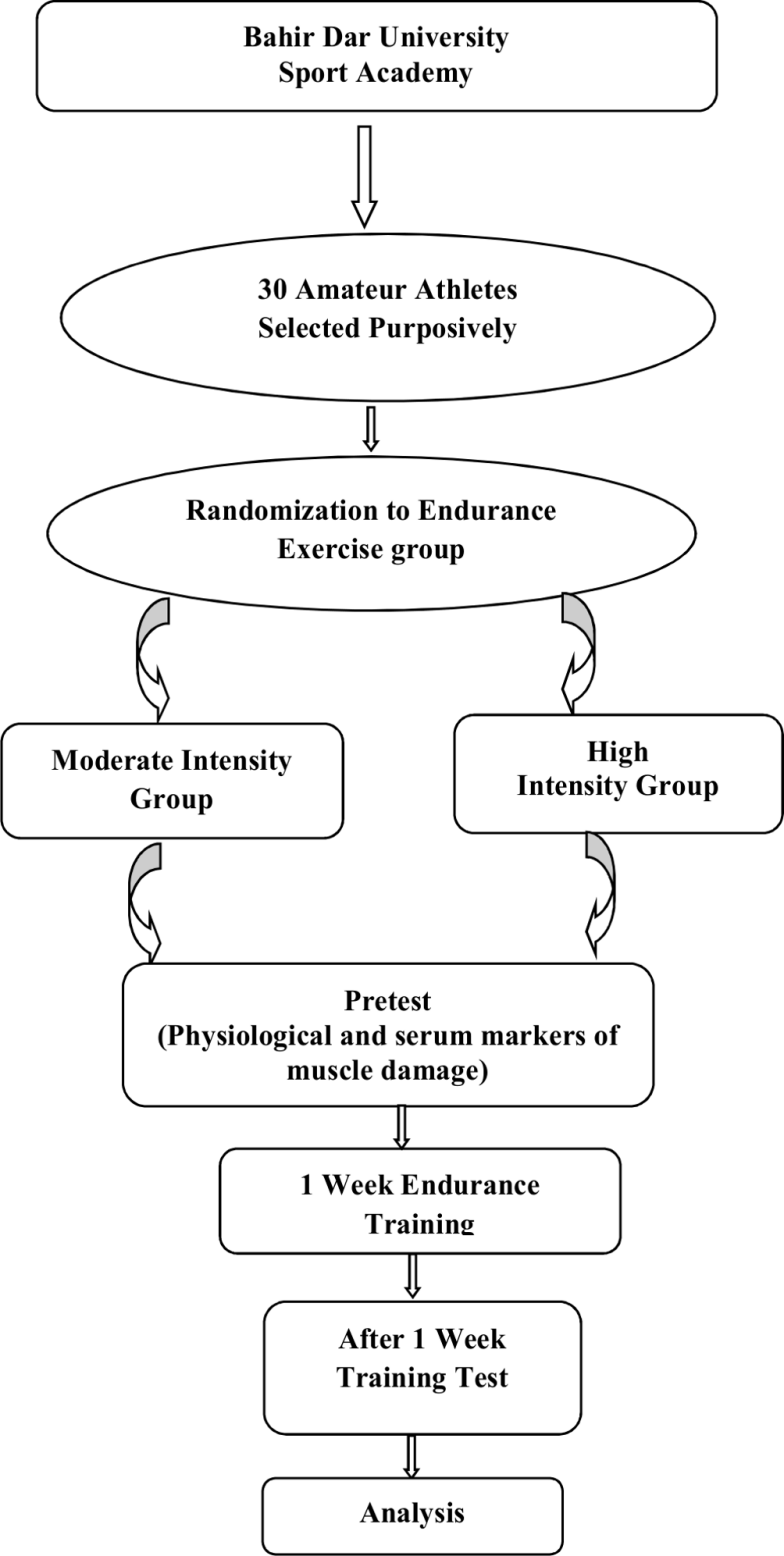
Flow chart for study design and data collection.

A randomised, parallel group study design was used in this study. Randomisation to endurance exercise is illustrated in (figure 1). Thus, 15 each of the 30 subjects participating in this study, was randomly assigned to two different intensity endurance exercise group. Pre-training and post-training, cardiorespiratory as well as cardiometabolic markers were assessed for both groups. Finally, post training within the group and between groups comparison was done.

### Training protocol and ethical approval

This field based study conducted based on the protocol specified here. Two professional athletics coaches are assigned one for the first and one for the second training groups. A 5 min warming up and stretching exercise given at the beginning of the training and 5 min of cooling down were also given at the end of each training time for both groups. Then continuous endurance running was given for 45 min at 60% to 70% HRmax for the first intensity training group and 30 min at 70% to 80% HRmax for the second intensity training group in the open field track per session for 3 days per week and it continued for 1 week. The training intensity was monitored with polar heart rate monitor throughout the entire session. The heart rate monitor adjusted to the designed percent maximum heart rate for both groups guided us whether it is bellow or above the designed % HRmax to adjust the heart rate drift throughout the training. We aimed to observe acute effects of the training on cardiometabolic markers as we are not expecting to get effective changes on cardiorespiratory markers. Both groups were instructed not to participate in other exercise training and to continue the usual habit of nutrition throughout the experiment.

Ethical approval was obtained from Institutional Research Ethics Review Committee of Mekelle University conformed to the 1975 Declaration of Helsinki. Written consent was delivered to the participants and they were informed about the objectives of the study. Participation in this study was purely a voluntary activity and their right not to participate was respected. Issues of confidentiality and anonymity were maintained too.

### Measurements of cardiorespiratory markers

Cardiorespiratory markers (VO_2_max, blood pressure and resting heart rate) were assessed just before beginning (pre-test) and after 1 week post-training test. VO_2_max of subjects was estimated using 3 min step test. Subjects stepped up and down a 16.5 inch stepping bench with a rate of 96 steps per minutes for 3 min in time of metronome beep sound. Five seconds after exactly 3 min pulse was taken from radial artery with heart rate monitor tied around the wrist. Finally maximal aerobic power (VO_2_max) was calculated using the equation VO_2_max=111.33 – (0.42×maximum pulse measured 5 s after stepping for 3 min)^24^ and reported in (ml/kg/min).

Systolic blood pressure (SBP) and diastolic blood pressure (DBP) was measured with Spengler digital electronic sphygmomanometer. The tourniquet of sphygmomanometer was tied on the wrist of the lower arm and inflated with air while subject seated. The result displayed from the indicator of sphygmomanometer was recorded in millimetre mercury. Mean arterial pressure (MAP) was computed using the following formula: MAP=DBP + 1/3 (SBP-DBP).^25^

Measurement of resting heart rate was done in the academy dormitory in the morning immediately after wake up using a heart rate monitor. The heart rate monitor sensor was tied on the radial artery of the hand and the subject laid down relaxed on supine position. After about 1 min, the heart rate monitor was started. The subject breathed calmly for 3 to 5 min without looking at the heart rate monitor. Then after 3 to 5 min the heart rate monitor was stopped. Finally the result of heart rate was checked and recorded.

### Assays of serum cardiometabolic markers

Blood samples of 5 mL were collected just before training (pre-test) from each participant. Then, post-training samples collected 4 hours after the final training session at the end of 1 week endurance training since the peak cardiometabolic markers are achieved 3 to 4 hours after training time.^26 27^ Blood was taken from an antecubital vein using Vacutainer Serum Separator Tube (SST) containing blood clotting accelerant gel. The serum was separated by centrifugation of the blood sample at 4000 rpm (revolution per minute) for 3 min and stored at −20°C until analysis. The levels of LDH were measured using a spectrophotometric assay with BS-2E chemistry analyser according to the guidelines of the International Federation of Clinical Chemistry (Beckman Coulter, Krefeld, Germany). While, the levels of cTnI and CK-MB was measured by chemiluminescence immunoassay using Maglumi 800 fully automated chemiluminescence immunoassay analyser via Shenzhen New Industries Biomedical Engineering Co, Ltd protocol.

### Data analysis

Finally, we analysed the result and compared the pre-training test to after 1 week tests as well as between group results. Analysis of the data obtained from the study inferential statistics was employed. To evaluate whether or not the data are normally distributed the Kolmogorov-Smirnov and Shapiro-Wilk^28 29^ normality test was applied. In case of normal distribution, Student’s paired t-test was used for an inter group (pre-post) comparison and to compare the effects of high and moderate intensity endurance training on physical performances and serum cardiac stress markers of athletes; one way between group analysis of variance was carried out using IBM-SPSS V.20. Each result of the data is expressed as mean±SD. All statistical inferences were seen at (p≤0.05) level of significance.

## Results

No between-group differences (age, height, body mass and body mass index) existed at baseline, so the groups were well matched at entry (table 1).

**Table 1:** General characteristics of participants

Parameters	Moderate intensity training group	High intensity training group	ANOVA
	M±SD	M±SD	P value
Age	19.27±1.28	19.47±1.30	0.67
Height	1.67±0.06	1.67±0.07	0.84
Body mass	53.87±4.76	53.96±5.67	0.964
BMI	19.39±1.49	19.31±1.41	0.881

ANOVA, analysis of variance; BMI, body mass index; M±SD, mean and standard deviation; P, probability.

Markers of cardiorespiratory and cardiometabolic response of pre and post 1 week endurance exercise training are presented in (table 2). Both intensity programme had no significant effect on markers of cardiorespiratory response within and between training groups (p>0.05). Mean values for cardiometabolic markers LDH (p=0.001), cTnI (p=0.001) and CK-MB (p=0.001) significantly increased at the end of 1 week within the group in both training intensity zones (p≤0.05). In the comparative analysis between training groups, there was no significant difference in markers of cardiometabolic response (p>0.05).

**Table 2:** A comparison of cardiorespiratory and cardiometabolic responses between and within the groups

Variables	Moderate intensity group		P value	High intensity group		between groups		P value
	Pre-test	Post-test		Pre-test	Post-test	P value	ANOVA	
	M±SD	M±SD		M±SD	M±SD		F	
Cardiorespiratory markers								
Systolic BP	120.5±8.3	119.4±10.3	0.762	113.5±5.5	114.5±6.5	0.644	7.449	0.01
Diastolic BP	74.1±8.5	77.5±9.80	0.242	73.5±8.7	74.1±6.9	0.828	0.29	0.866
MAP	89.5±7.1	91.6±9.7	0.454	86.8±6.5	87.3±4.8	0.759	2.275	0.143
RHR	75.9±8.3	71.7±0.6	0.250	71.2±8.8	71.7±7.0	0.872	2.235	0.146
VO_2_max	60.7±4.5	62.2±4.7	0.345	60.4±4.5	60.8±4.2	0.082	0.46	0.831
Cardiometabolic markers								
LDH	136±38.4	245.8±102.1	0.002	178.9±52	297.5±10	0.001	1.961	0.172
cTnI	0.10±0.11	0.44±0.22	0.0001	0.12±0.12	0.59±0.32	0.0001	2.482	0.126
CK-MB	1.12±0.97	3.39±10	0.0002	1.71±1.4	4.1±1.6	0.00001	1.764	0.195

ANOVA, analysis of variance; BP, blood pressure; CK-MB, creatine kinase myocardial band; cTnI, cardiac troponin I; LDH, lactate dehydrogenase; MAP, mean arterial pressure; M±SD, mean and standard deviation; RHR, resting heart rate; VO_2_max, maximum oxygen consumption.

## Discussion

This study compared the effects of short-different intensity endurance training on markers for cardiorespiratory and cardiometabolic responses in young moderately trained athletes. Our main finding observed no major difference in markers for cardiorespiratory and cardiometabolic markers between training groups. However, a significant difference was observed in markers for cardiometabolic and but not cardiorespiratory within training groups.

Consistent with our finding, previous studies reported no difference in markers for cardiorespiratory responses between and within intensity training groups.^14–16^ Furthermore, a study by Gormley *et al*^30^ administered endurance training under various intensities and reported no significant difference in markers for cardiorespiratory responses, between groups. Indeed, a meta-analysis, that coded training status and similar training intensities in sedentary subjects, reported that every training intensity from below lactate threshold to above 4 mmol·L^-1^ blood lactate concentration improved performance, with no significant difference between the intensities. Thus, the reason for no observed differences could be due to administering endurance training either at or below lactate threshold that equally evoke a similar physiological response in amateur athletes.^31 32^ Therefore, if the main goal of endurance exercise is to improve markers for cardiorespiratory responses for amateur athletes, it would be better done under moderate intensity training similar to 60% to 70% HRmax. Considering that younger athletes have been reported to possess reduced arterial stiffness and might respond to short-term endurance exercise.^33^

Studies by Peters *et al*^34^ and Baker *et al*^35^ reported that moderate and high intensity endurance training corresponds to the designed intensities in our modified protocol does not lead to elevations in serum markers of cardiometabolic responses within the groups in amateur athletes. However, elevation in serum markers of cardiometabolic responses within groups has been reported^10 13 35–37^ and our findings are in agreement. Besides, a study by Bauer *et al*^38^ and Callegari *et al*^39^ also reported an elevation in serum markers of cardiometabolic responses in elite athletes. Interestingly, exercise is possibly the only documented cause of increases serum markers of cardiometabolic responses such as cardiac troponin that is not associated with adverse clinical outcome. This has led to the hypothesis that an elevation in serum markers of cardiometabolic responses is part of a normal physiological response to exercise and does not represent irreversible cell death. It is important to highlight that, the elevation in serum markers of cardiometabolic responses is often associated with younger age and might reflect poor adaptation to endurance training.^35^ Since, poor adaptation to endurance training in young athletes has been related to increased muscle and cartilage inflammation^3^ that may result to increased serum cardiometabolic markers. Consequently, our finding and others^39^ did not observe differences in serum markers of cardiometabolic response between short-term different intensity endurance training groups. Whether elevation of serum markers cardiometabolic responses of young athletes might indicate poor or better adaptation to endurance training needs further investigation.

Exercise intensity at the boundary of moderate-to-high intensity^40^ can be maintained for a prolonged period of time and is therefore very attractive to untrained individuals explained as decreased cardiac markers as maximum oxygen consumption increased after training adaptation.^41^ Our findings imply that training intensity zones 60% to 70% HRmax or 70% to 80% HRmax, may induce similar effects on serum markers for cardiometabolic and cardiorespiratory in amateur athletes. However, different training duration is the limitation of this study. It is likely that in young athletes these events are a normal, potentially adaptive, process associated with the work of prolonged exercise. Further study is required, whether these changes are associated with tissue damage and what the long-term consequences of such maladaptation might be.

## Conclusion

One week 60% to 70% HRmax and 70% to 80% HRmax intensity zones induces similar acute effects on markers for cardiometabolic and cardiorespiratory responses in amateur athletes between groups and significant elevations of cardiometabolic markers within the groups.
